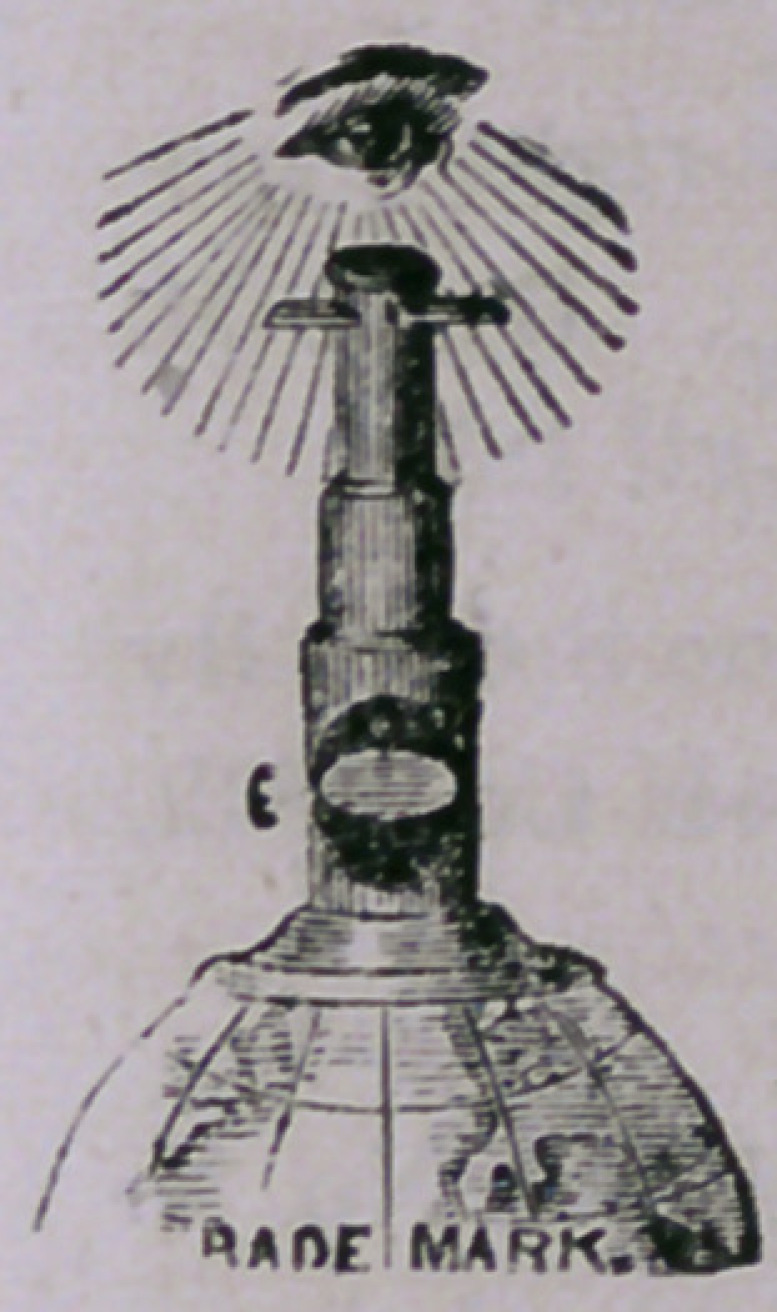# The Globe Microscope

**Published:** 1872-06

**Authors:** 


THE GLOBE MICROSCOPE.—We have received one of these little miscroscopes and are truly surprised at the magnifying power which they possess.

The above cut will represent to our readers pretty fairly the character and style of the microscope. Although not one of the kind which would be made useful by the professional microscopist, yet it opens up to the eye of the common observer a wide field of research in the minute world, and can not fail to be a source of amusement and instruction in studying the forms of world of minute life. They are manufactured by Geo. Mead & Co., of Racine, Wis.

## Figures and Tables

**Figure f1:**